# N_2_-Fixing *Fontibacillus forbon* sp. nov., a Novel Species from the Plant Rhizosphere

**DOI:** 10.3390/microorganisms14010049

**Published:** 2025-12-25

**Authors:** Rui Hu, Yimin Shang, Weilong Zhang, Chengao Song, Renzong Wang, Sanfeng Chen

**Affiliations:** 1 College of Biological Sciences, China Agricultural University, Beijing 100193, China; 2 Key Laboratory of Arable Remediation Technology (Central China), Ministry of Agriculture and Rural Affairs, Forbon Technology Co., Ltd., Wuhan 430206, China

**Keywords:** *Fontibacillus forbon* sp. nov., genomic characteristics, phylogenetic analysis, nitrogen-fixing bacterium

## Abstract

N_2_-fixing bacteria have great potential to be used as biofertilizer in agriculture to promote plant growth via nitrogen fixation. In this study, a novel species *Fontibacillus forbon* sp. nov., with strain BL-9^T^ as the type strain, was isolated from the rhizosphere of *Fraxinus chinensis*. Strain BL-9^T^ was able to fix nitrogen and grow on nitrogen-free medium. Phylogenetic analysis of 16S rRNA gene revealed that strain BL-9^T^ was most closely related to *Fontibacillus phaseoli* BAPVE7B (98.03%), followed by *Fontibacillus solani* A4STR04 (96.72%), *Fontibacillus panacisegetis* (96.6%), *Paenibacillus vini* (96.6%), and *Paenibacillus segetis* DB13260 (96.57%). The phylogenomic tree supported that strain BL-9^T^ was most closely related to *F. phaseoli* BAPVE7B. The digital DNA-DNA hybridization (dDDH) and average nucleotide identity (ANI) between strain BL-9^T^ and its closely related type strain, *F. phaseoli* BAPVE7B, were 42.5% and 90.94%, respectively, which were below the values (70% for dDDH and 95% for ANI) for species discrimination. The DNA G+C content of strain BL-9^T^ was 49.7%. The genome of strain BL-9^T^ had a *nif* (*ni*trogen *f*ixation) gene cluster containing 10 genes (*nifB nifH nifD nifK nifE nifN nifX orf1 hesA nifV*). The predominant fatty acid was anteiso-C15:0, the major menaquinone was MK-7, and the major polar lipid was diphosphatidylglycerol. Strain BL-9^T^ and its closely related species of *Fontibacillus* had some common and distinguished physiological characteristics. Based on genomic, phylogenetic, chemotaxonomic, and phenotypic features, strain BL-9^T^ represents a novel species of the genus *Fontibacillus.* The name proposed for this species is *Fontibacillus forbon* sp. nov., with the type strain BL-9^T^.

## 1. Introduction

The family *Paenibacillaceae* of the order *Bacillales* was created by De Vos in 2009 [1]. Most of the members of the family *Paenibacillaceae* have rod-shaped cells with different physiological characteristics, such as being Gram-positive or Gram-negative, having ellipsoidal endospores in swollen sporangia, and being aerobic or facultatively anaerobic [1]. At the time of writing, the family *Paenibacillaceae* currently comprises 19 recognized genera: *Ammoniibacillus* [2], *Ammoniphilus* [3], *Aneurinibacillus* [4], *Brevibacillus* [4], *Chengkuizengella* [5], *Cohnella* [6], *Ferviditalea* [7], *Fontibacillus* [8], *Gordoniibacillus* [9], *Gorillibacterium* [10], *Insulibacter* [11], *Longirhabdus*, *Marinicrinis* [12], *Oxalophagus* [13], *Paenibacillus* [14], *Paludirhabdus* [15], *Saccharibacillus* [16], *Thermobacillus* [17], and *Xylanibacillus* [18] (https://lpsn.dsmz.de/family/paenibacillaceae, accessed on 12 November 2025).

Within the family *Paenibacillaceae,* the genus *Fontibacillus* is very closely related to the genus *Paenibacillus*. The genus *Paenibacillus* with *Paenibacillus polymyxa* as the type species was created in 1993, and at that time it encompassed eleven species, three of which, *Paenibacillus polymyxa*, *Paenibacillus azotofixans,* and *Paenibacillus macerans,* were N_2_-fixing species [14]. The genus *Paenibacillus* has developed as a large genus comprising 400 species (https://lpsn.dsmz.de/genus/paenibacillus, accessed on 12 November 2025). Many novel species and strains with N_2_-fixing ability have been described [19,20,21]. A *nif* (*ni*trogen *f*ixation) gene cluster composed of 9–10 genes [*nifB nifH nifD nifK nifE nifN nifX hesA* (*orf1*) *nifV*] encoding Mo-nitrogenase is conserved in N_2_-fixing *Paenibacillus* strains [19,20,21,22]. In addition, some N_2_-fixing *Paenibacillus* species have V-nitrogenase or Fe-nitrogenase [19]. Some members of *Paenibacillus* can promote plant growth via nitrogen fixation [23]. Inoculation of N_2_-fixng *Paenibacillus triticisoli* BJ-18 as a bio-fertilizer resulted in changes to the microbial community structure in the rhizosphere of wheat in fields [24].

Genus *Fontibacillus* with *F. aquaticusas* as the type species was created in 2010, based on chemotaxonomic characteristics and the 16S rRNA gene [8]. Compared to *Paenibacillus*, the genus *Fontibacillus* is a small genus which includes five recognized species. *F. aquaticus* gen. nov., sp. nov., was isolated from a warm spring [8]. *Fontibacillus panacisegetis* sp. nov. was isolated from soil of a ginseng field [25]. *Fontibacillus phaseoli* sp. nov. was isolated from *Phaseolus vulgaris* nodules [26]. *Fontibacillus solani* sp. nov. was isolated from potato root [27]. *Fontibacillus pullulanilyticus* sp. nov. was isolated from soil [28]. Among the five *Fontibacillus* species, only *F. phaseoli* is a N_2_-fixing bacterium which has Mo-nitrogenase encoded by 10 genes (*nifB nifH nifD nifK nifE nifN nifX hesA orf1 nifV*).

Nitrogen (N) is the most important nutrient for plant growth. Growth of non-legume plants, such as rice wheat and maize, depends highly on chemical N fertilizers. However, production and over-application of chemical N fertilizers results in economic costs and environmental pollution. One approach to reduce use of N fertilizers is inoculation of non-legume plants with N2-fixing bacteria (biofertilizer), and another approach is the direct transfer of *nif* genes into cereal crops so that they can fix their nitrogen by using synthetic biology. Thus, it is necessary to isolate the novel N_2_-fixing microorganisms for application as biofertilizer and in engineering *nif* genes into non-legume plants.

In this study, 118 soil samples were taken from the rhizospheres of 17 plants, including rice, maize, wheat, oat, cowpea, onion, cabbage, spinach, rapeseed, eggplant, coriander, cluster mallow, fragrant plantain lily, peppermint, tall fescue, poplar, and ash tree (*Fraxinus chinensis*) in different regions of China. These soil samples were individually suspended in sterile water, and then these suspensions were individually spread on nitrogen-free medium agar plates for the growth of bacterial colonies. Twenty-four strains with the *nifH* gene encoding Fe protein of nitrogenase were obtained by screening 3200 bacterial colonies by PCR amplification using the *nifH* gene as a probe. Analysis of 16S rRNA gene revealed that among the 24 strains, only strain BL-9^T^, isolated from the rhizosphere of *Fraxinus chinensis*, belongs to *Fontibacillus* genus and the other 23 strains are members of the *Klebsiella* and *Paenibacillus* genera. Based on genomic, phylogenetic, chemotaxonomic, and phenotypic features, strain BL-9^T^ is demonstrated to be a novel species of the genus *Fontibacillus* and the name proposed for this species is *Fontibacillus forbon* sp. nov. As we know, *Fontibacillus forbon* sp. nov is the second N_2_-fixing species within the *Fontibacillus* genus. The N_2_-fixing *Fontibacillus* species might have potential application as biofertilizer just as some N_2_-fixing *Paenibacillus* species did.

## 2. Materials and Methods

### 2.1. Isolation of Strains

N_2_-fixing microorganisms were isolated by using nitrogen-free medium which was composed of 0.1 g NaCl, 0.01 g FeCl_3_, 0.2 g MgSO_4_·7H_2_O, 0.002 g Na_2_MoO_4_, 0.1 g K_2_HPO_4_, 0.4 g KH_2_PO_4,_ and 20 g sucrose per liter [21]. A total of 118 soil samples were taken from the rhizospheres of 17 plants, including rice, maize, wheat, oat, cowpea, onion, cabbage, spinach, rapeseed, eggplant, coriander, cluster mallow, fragrant plantain lily, peppermint, tall fescue, poplar, and ash tree (*Fraxinus chinensis*) in Hebei province, Henan province, Jiangsu province, Sichuan province and Beijing suburbs, China. Each soil sample was suspended in sterile water, and 100 μL suspension was spread on nitrogen-free medium agar plates. After incubation at 30 °C for 3 days, single colonies were identified by PCR amplification with the *nif* gene probe.

Strain BL-9^T^ was isolated from the rhizosphere of *Fraxinus chinensis* in Haidian District of Beijing, China (39°57′52.84′′ N 116°17′52.84′′ E). The soil type is sandy loam, and the organic matter content is about 18 g/kg with pH 6.0–8.0.

### 2.2. Sequence Analysis of nifH and 16S rRNA Genes and Construction of Phylogenetic Trees

NifH protein encoded by the *nifH* gene is a structural unit of nitrogenase and *nifH* is a key gene used for identification of N_2_-fixing microorganisms. The *nifH* gene of strain BL-9^T^ was PCR amplified with primers *nifH-*P1 (5′-GGCTGCGATCCVAAGGCCGAYTCVACCCG-3′) and *nifH-*P2 (5′-CTGVGCCTTGTTYT CGCGGATSGGCATGGC-3′) [21]. The 16S rRNA gene of strain BL-9^T^ was PCR-amplified with primers 16S P1 (5′-AGAGTTTGATCCTGGCTCAGAACGAACGCT-3′) and 16S P2 (5′-TACGGCTACCTTGTTACGACTTCACCCC-3′) [29]. The PCR-amplified *nifH* and 16S rRNA gene products were sequenced. The sequences of the *nifH* gene and 16S rRNA gene were analyzed on NCBI (https://blast.ncbi.nlm.nih.gov, accessed on 12 November 2025). The phylogenetic tree was constructed with the maximum likelihood (ML) in the software MEGA7 [30]. Bootstrap analysis with 500 replicates was performed, and bootstrap values were calculated to evaluate the confidence levels of tree branches.

### 2.3. Genome Sequencing

The genomic DNA of strain BL-9^T^ was extracted using the TIANamp Bacteria DNA Kit (DP302-02) made by TIANGEN BIOTECH Co., Ltd., Beijing, China. Genome sequencing was performed in Biomarker Technologies, Beijing, China by using the Illumina PE150 platform. Fragments below 500 bp were filtered out, and contaminated samples were further decontaminated. For genome assembly, the filtered reads were assembled by Spades v3.6.2 software. The assembled genome was then evaluated, statistically analyzed, and subject to subsequent gene prediction. The GeneMarkS software (Version 4.17) was employed to predict protein-coding genes in the sequenced genome [31]. The protein sequences of predicted genes were aligned with various functional databases using Diamond (e value ≤ 1 × 10^−5^). Genomic assembly metrics of strain BL-9^T^ are shown in Table 1.

### 2.4. Genomic Feature Analysis

Digital DNA–DNA hybridization (dDDH) values were computed using GGDC (Genome-to-Genome Distance Calculator) [32] at https://ggdc.dsmz.de/ggdc.php, accessed on 12 November 2025. The whole-genome similarity was assessed using the Average Nucleotide Identity (ANI) tool with the OrthoANIu algorithm (https://www.ezbiocloud.net/tools/ani, accessed on 12 November 2025) [33]. Genome sequences of reference strains were from https://www.ncbi.nlm.nih.gov/datasets/genome/, accessed on 12 November 2025.

For construction of a genome based phylogenetic tree, the 92 single-copy core genes were extracted from the genome sequences using the UBCG program [34]. Then, the phylogenomic tree was reconstructed using IQ-TREE v2.0.7 software [35] based on the concatenated sequence dataset with 1000 bootstrap replicates.

### 2.5. Nitrogenase Activity Assay

The acetylene reduction assay was used to measure the nitrogenase activity of strain BL-9^T^ as described previously [20,36], and *Paenibacillus polymyxa* WLY78 was used as a positive control.

Each strain of BL-9^T^ and *P. polymyxa* WLY78 was anaerobically grown in nitrogen-deficient medium supplemented with 2 mM glutamate to a final OD_600_ of 0.2–0.4. Then, 1 mL of culture was transferred to a 25 mL test tube, and then the test tube was sealed with a robber stopper. The headspace in the tube was evacuated and replaced with argon gas. After incubating the cultures for 6–8 h at 30 °C with shaking, C_2_H_2_ (10% of the headspace volume) was injected into the test tubes. Incubating for another 3 h, 100 μL gas was withdrawn from the test tube through the rubber stopper with a gas tight syringe and then injected into gas chromatograph to quantify C_2_H_4_ production. The nitrogen activity was expressed in nmol C_2_H_4_/mg protein/h. All treatments were in three replicates.

### 2.6. Chemotaxonomic Characterization

Whole-cell fatty acids, polar lipids, and respiratory quinones were analyzed by Preservation Center of China Agricultural Microbial Strains in Chinese Academy of Agricultural Sciences, Beijing, China. Polar lipid was extracted according to the method described by Minnikin et al. [37] and was identified by two-dimensional TLC (Thin Layer Chromatography). Analysis of compositions of cellular fatty acids was performed by the method described by Komagata and Suzuki [38] using the Sherlock Identification System (MIDI) [39]. Cellular menaquinones and isoprenoid quinones were extracted and analyzed using HPLC (High Performance Liquid Chromatography) [40].

### 2.7. Morphological, Physiological, and Biochemical Analysis

Strain BL-9^T^ and reference strains were routinely grown on LB agar at 30 °C for 2–3 days.

Cell morphology was observed by scanning electrical microscopy (SEM). Physiological and biochemical tests, such as starch hydrolysis, nitrate reduction, and NaCl tolerance, were performed as described by Zhang et al. [21].

Gram staining of strain BL-9^T^ grown on LB medium for 14 h was performed with *E. coli* and *Bacillus subtilis* as controls. Bacterial cells were spread on a glass slide and dried in air. Then, crystal purple solution was added to smear on the glass slide. After 1 min, the smear was rinsed with distilled water. Iodine solution was added to the smear for 1 min and then the smear on the glass slide was rinsed with distilled water. 95% ethanol was added to the smear on the glass slide for 20 s. Sarranine solution was added to the smear on the glass slide for 1 min and the smear on the glass slide was rinsed with distilled water. Finally, bacterial cells were observed under a microscope. All treatments were in three replicates.

## 3. Results and Discussion

### 3.1. Isolation of N_2_-Fixing Microorganisms

A total of 118 soil samples were taken from the rhizospheres of 17 plants, including rice, maize, wheat, oat, cowpea, onion, cabbage, spinach, rapeseed, eggplant, coriander, cluster mallow, fragrant plantain lily, peppermint, tall fescue, poplar, and ash tree (*Fraxinus chinensis*) in different regions of China. These soil samples were individually resuspended in sterile water, and these resuspensions were individually spread on nitrogen-free medium for the growth of bacterial colonies. Twenty-four strains with *nifH* gene encoding Fe protein of nitrogenase were obtained by screening 3200 bacterial colonies using PCR amplification with the *nifH* gene as a probe. Analysis of the 16S rRNA gene revealed that among the 24 strains, only strain BL-9^T^, isolated from the rhizosphere of *Fraxinus chinensis*, belongs to *Fontibacillus* genus, and the other 23 strains belong to *Klebsiella* and *Paenibacillus* genera. Strain BL-9^T^ was then selected to be further investigated.

### 3.2. Phylogenetic Analysis of 16S rRNA Gene

**Figure 1:**
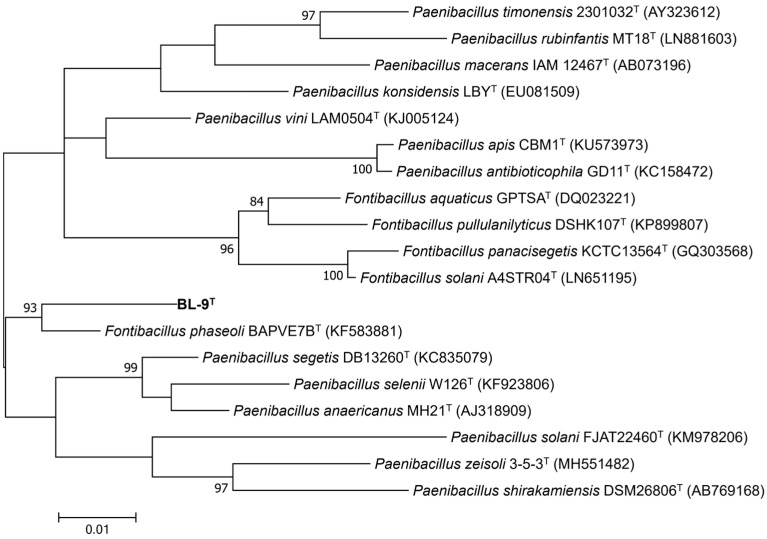
Maximum-likelihood phylogenetic tree based on nearly complete 16S rRNA gene sequences showing the relationship between strain BL-9T and its closely related members of the *Fontibacillus* and *Paenibacillus* genera.

Homology comparison of the 16S rRNA gene sequence of strain BL-9^T^ with those held in the GenBank database revealed that strain BL-9^T^ had high similarity to members of both *Fontibacillus* and *Paenibacillus* genera. Strain BL-9^T^ showed the highest sequence similarity with *Fontibacillus phaseoli* BAPVE7B (98.03%), followed by *Fontibacillus solani* A4STR04 (96.72%), *Fontibacillus panacisegetis* (96.6%), *Paenibacillus vini* (96.6%), *Paenibacillus anaericanus* (96.6%), and *Paenibacillus segetis* DB13260 (96.57%). A 98.65% similarity of 16S rRNA gene sequences is the threshold to differentiate bacterial species [41]. The phylogenetic tree based on the 16S rRNA gene sequences exhibited that strain BL-9^T^ formed a distinct monophyletic group with *F. phaseoli* BAPVE7B^T^, supported by a high bootstrap value of 93% (Figure 1). These results indicate that strain BL-9^T^ is a novel species within the genus *Fontibacillus*.

### 3.3. Genomic Features and Phylogenomic Tree

Strain BL-9^T^ was genome-sequenced to evaluate its genome features. The genome size of strain BL-9^T^ is 5.5 Mb with 5081 genes. There are 91 tRNA genes and a 16S rRNA gene. The DNA G+C content of strain BL-9^T^ is 49.7% (Table 2). Whereas its closest relative strain *F. phaseoli* has a genome size of 5.4 Mb with 5071 genes. The DNA G+C content of *F. phaseoli* is 49.5%. The genome sequence of strains BL-9^T^ was deposited in the GenBank under accession number GCA_046559655.1 (Table 1).

**Figure 2:**
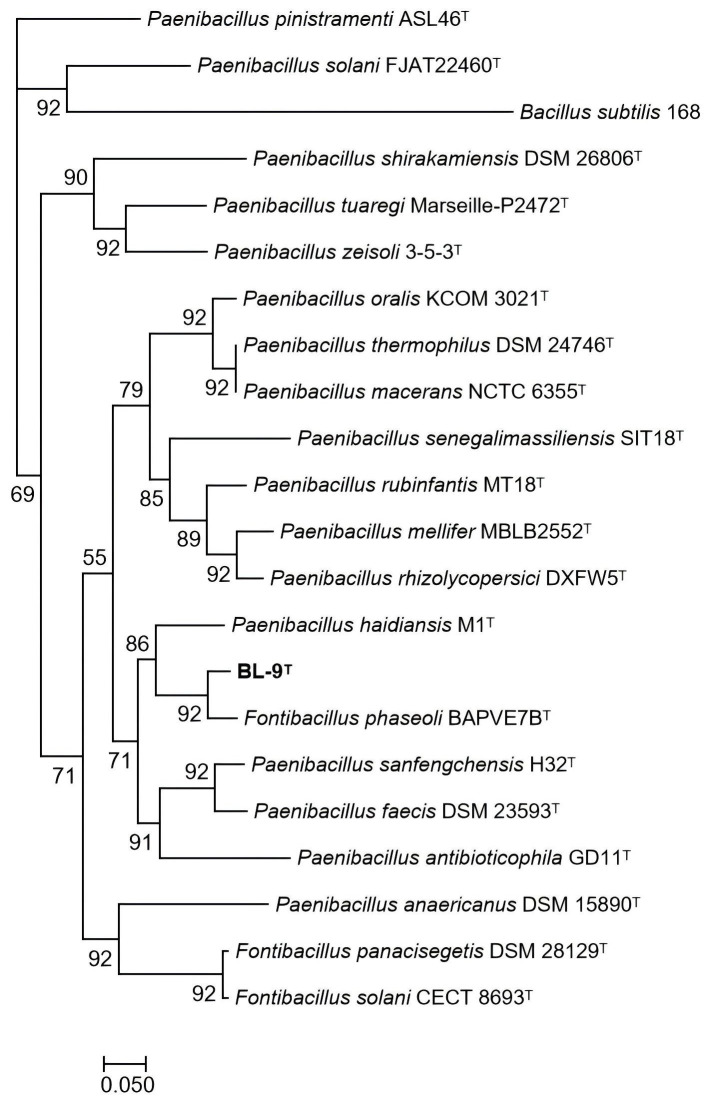
Phylogenomic tree of the novel strain BL-9^T^ and members of the genera *Fontibacillus and Paenibacillus* inferred using ML algorithms based on the concatenated alignment of 92 core genes. Bootstrap values (>50%) based on 1000 replicates are shown at the branch nodes. *Bacillus subtilis* 168 was used as the outgroup. Bar, 0.1 substitutions per nucleotide position.

The phylogenomic tree (Figure 2), based on 92 single-cope core genes, demonstrated that strain BL-9^T^ was clustered together with *F. phaseoli* BAPVE7B^T^, consistent with the phylogenetic analysis of the 16S rRNA gene described above.

It is recognized that ANI and dDDH for the species threshold are 95% and 70%, respectively [33,42,43]. ANI and dDDH values were obtained by comparing genome sequence of strain BL-9^T^ with those of the closely related species of *Fontibacillus* and *Paenibacillus* genera (Table 3). The highest ANI (42.5%) and the highest dDDH (90.94%) were between strain BL-9^T^ and reference strain *F. phaseoli* BAPVE7B. The ANI and dDDH values between strain BL-9^T^ and its closely related species of *Fontibacillus* and *Paenibacillus* genera were 69.72–82.32% and 18.0–25.7%, respectively (Table 3). These genomic relatedness data of strain BL-9^T^ are below the thresholds of ANI (95.0%) and dDDH (70.0%), indicating that strain BL-9^T^ is a novel species of *Fontibacillus* genus.

### 3.4. Nitrogen Fixation (nif) Genes and Nitrogenase Activity

Nitrogenase activity was measured by the acetylene reduction assay as described in the methods section. Strain BL-9^T^ exhibited nitrogenase activity with 4802 (nmol C_2_H_4_/mg protein/h), while *P. polymyxa* WLY78 (positive control) had nitrogenase activity with 3479 (nmol C_2_H_4_/mg protein/h). The results are consistent with the reports that nitrogenase activities exhibited variation among different N_2_-fxing strains [20,21].

The *nifH* gene encoding subunit of nitrogenase is highly conserved among N_2_-fixing organisms and it is used as an indicator for identifying nitrogen-fixing bacteria. The *nifH* gene of strain BL-9^T^ exhibited the highest similarity with *Paenibacillus abekawaensis* MG1 (74.6%), followed by *Paenibacillus stellifer* DSM14472 (73.4%), and *Paenibacillus durus* DSM1735 (73.1%). The *nifH* gene of strain BL-9^T^ also had high similarity with those of other N_2_-fixing bacteria (e.g., *Methylococcus capsulatus*, *Rhodobacter sphaeroides*, *Bradyrhizobium amphicarpzeae*). The phylogenetic tree based on *nifH* gene sequences showed that strain BL-9^T^ is clustered together with the N_2_-fixing *Paenibacillus* species (Figure S1).

**Figure 3:**
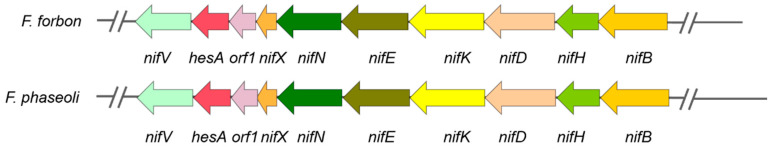
*nif* (*ni*trogen *f*ixation) gene organization from the novel species *Fontibacillus forbon* BL-9^T^ and *F. phaseoli* BAPVE7B.

Genome sequence analysis showed that strain Bl-9^T^ has a Mo-nitrogenase encoded by a *nif* gene cluster containing 10 genes (*nifB, nifH, nifD, nifK, nifE, nifN, nifX, orf1, hesA,* and *nifV*), just as observed in *F. phaseoli* BAPVE7B (Figure 3). The *nif* gene cluster of strain Bl-9^T^ has 91.5% identity with that of *F. phaseoli* BAPVE7B (Table S1). These data supported that strain BL-9^T^ is a N_2_-fixing bacterium which was isolated from the rhizosphere of *Fraxinus chinensis*, whereas *F. phaseoli* BAPVE7B was isolated from *Phaseolus vulgaris* nodules [26]. In N_2_-fixing *Paenibacillus* spp., some species (e.g., *P. ploymyxa*, *P. beijingensis*, *P. massiliensis*) have nine genes (*nifB, nifH, nifD, nifK, nifE, nifN, nifX, hesA* and *nifV)*, while some species (e.g., *Paenibacillus sabinae*, *Paenibacillus forsythiae*, *Paenibacillus graminis*) have ten genes, which have an additional *orf1* [22,26].

### 3.5. Morphological and Physiological Characteristics

**Figure 4:**
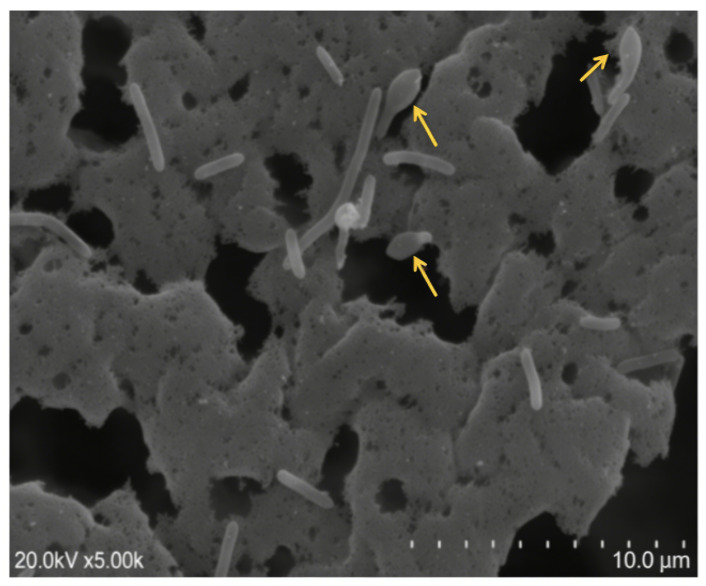
Morphology of vegetative cells and endospores of the novel strain BL-9^T^ observed under scanning electron microscopy. Arrow in yellow indicates sporangia containing spore.

Strain BL-9^T^ was Gram-negative, facultatively anaerobic, motile, and rod-shaped. Our results are in agreement with the reports that some species of the order *Bacillales* are Gram-variable, although most of the species from the order *Bacillales* are Gram-positive. For example, *Paenibacillus favisporus* was Gram-variable [44]. Scanning electron microscopy of strain BL-9^T^ grown on LB agar for three days revealed ellipsoidal spores in swollen sporangia (Figure 4).

Strain BL-9^T^ and the reference strains *F. phaseoli* BAPVE7B^T^, *F. solani* A4STR04^T^, and *P. segetis* DB13260^T^ were tested for a range of physiological and biochemical characteristics. Strain BL-9^T^, *F. phaseoli* BAPVE7B^T^, and *F. solani* A4STR04^T^ exhibited positive nitrate reductase activity and starch hydrolysis, while *P. segetis* DB13260^T^ had negative nitrate reductase activity and did not hydrolyze starch. Strain BL-9^T^ utilized fructose, glucose, galactose, maltose, mannitol, sorbitol, sucrose, and lactose to produce acid, while *P. segetis* DB13260^T^ utilized all of these compounds except for inositol. However, reference strain *F. phaseoli* BAPVE7B^T^ only utilized glucose, galactose, maltose, sucrose, and lactose to produce acid. Reference strain *F. solani* A4STR04^T^ utilized fructose, maltose, and lactose, and utilized weakly galactose and sucrose. Differentiatial phenotypic characteristics among strain BL-9^T^ and reference strains are summarized in Table 4.

### 3.6. Chemotaxonomic Characteristics

Chemotaxonomic features of strain BL-9^T^ were determined according to the previously described methods [22,41,42,43]. Analysis of the cellular fatty acid revealed that anteiso-C15:0 is the major fatty acid component of strain BL-9^T^ (55.14%) (Table 3). Anteiso-C15:0 is also the predominant fatty acid for its reference strains *F. phaseoli* BAPVE7B^T^ (53.1%), *F. solani* A4STR04^T^ (61.5%), and *P. segetis* DB13260^T^ (43.9%). The fatty acid contents showed significant variation among strain BL-9^T^ and reference strains, although anteiso-C15:0 is the major fatty acid for strain BL-9 and its reference strains (Table 5). These data indicate that strain BL-9^T^ is distinguished from other members of both genera *Fontibacillus* and *Paenibacillus.*

Strain BL-9^T^ contains the major polar lipids, including DPG (diphosphatidylglycerol), APL (aminophospholipids), PG (phosphatidylglycerol), APGL (unidentified aminophosphoglycolipid), and unidentified phospholipids (PL2, PL3 and PL4) (Figure S2). The results showed that the polar lipid profiles of strain BL-9^T^ are similar to those of *Fontibacillus* spp. (e.g., *F. aquaticus* and *F. phaseoli*) [8,19], supporting that strain BL-9^T^ is a member of the *Fontibacillus* genus.

The major respiratory quinone component of strain BL-9^T^ is menaquinone-7 (MK-7) (Figure S3). MK-7 is also the only menaquinone in the *Fontibacillus* species, such as *F. aquaticus* and *F. panacisegetis* [8,23]. The data support that strain BL-9^T^ is a member of the *Fontibacillus* genus.

## 4. Conclusions

A novel species *Fontibacillus forbon* sp. nov., with strain BL-9^T^ as the type strain was isolated from the rhizosphere of *Fraxinus chinensis*. Strain BL-9^T^ is a facultatively anaerobic, rod-shaped, endospore-forming, and motile bacterium. Strain BL-9^T^ was able to fix nitrogen and grew on nitrogen-free medium. Genome of strain BL-9^T^ has a *nif* (*ni*trogen *f*ixation) gene cluster containing 10 genes (*nifB nifH nifD nifK nifE nifN nifX orf1 hesA nifV*). DNA G+C content of strain BL-9^T^ is 49.7%. The predominant fatty acid is anteiso-C15:0, the major menaquinone is MK-7, and the major polar lipid is diphosphatidylglycerol. Chemotaxonomic analyses demonstrated that strain BL-9^T^ and species of the genus *Fontibacillus* have common features, with anteiso-C15:0 as the predominant fatty acid, MK-7 as the major menaquinone, and diphosphatidylglycerol as the major polar lipid. Strain BL-9^T^ and its closely related species of *Fontibacillus* have some common and distinguished physiological characteristics. Phylogenies, based on the 16S rRNA gene and core genome, revealed that strain BL-9^T^ was most closely related to *Fontibacillus phaseoli* BAPVE7B. However, the digital DNA-DNA hybridization (dDDH) and average nucleotide identity (ANI) between strain BL-9^T^ and its closely related type strain *F. phaseoli* BAPVE7B were 42.5% and 90.94%, respectively, indicating that strain BL-9^T^ represents a novel species of the genus *Fontibacillus.* The name proposed for this species is *Fontibacillus forbon* sp. nov., with the type strain BL-9^T^ (=GDMCC 1.5526^T^ = JCM 37804^T^).

## 5. Description of *Fontibacillus forbon* sp. nov

*Fontibacillus forbon* (forbon, named after Forbon Technology Co., Ltd., Wuhan, China).

Cells are Gram-negative, facultatively anaerobic, rod-shaped, and motile. An ellipsoidal spore is formed in swollen sporangia. The colonies on the LB medium are cream white, convex, and circular, with a diameter of 1.0–2.0 mm. Cells grow at 25–35 °C, with optimum growth at 26 °C. The pH range for growth is a pH of 6.0–8.0 (optimum pH 7.0). The optimum concentration of NaCl for growth is 1.0%. The various substrates: D-fructose, D-galactose, D-glucose, D-xylose, lactose, maltose, sucrose, inositol, D-mannitol, and D-sorbitol, are utilized. Nitrate is reduced to nitrite. Starch is hydrolyzed. The predominant fatty acid is anteiso-C15:0. The major polar lipids are DPG (diphosphatidylglycerol), APL (aminophospholipids), PG (phosphatidylglycerol), and APGL (unidentified aminophosphoglycolipid). The major menaquinone is MK-7. Strain BL-9^T^ exhibits nitrogenase activity and has a *nif* gene cluster composed of 10 genes (*nifB*, *nifH*, *nifD*, *nifK*, *nifE*, *nifN*, *nifX*, *orf1*, *hesA*, *nifV*). The genome size is 5.5 Mb, and the G+C content is 49.7%.

The type strain BL-9^T^ (=CGMCC 1.5526^T^ = JCM 37804^T^) was isolated from the rhizosphere soil of *Fraxinus chinensis* in the Haidian District of Beijing, China. The GenBank accession numbers for the 16S rRNA sequence and for the genome sequence are PQ803957.1. and GCA_046559655.1., respectively. The type strain BL-9^T^ was deposited in the Japan Collection of Microorganisms with No. JCM 37804^T^ and in the Chinese Guangdong Microbial Culture Collection Center with No. CGMCC 1.5526^T^.

## Figures and Tables

**Table 1:** Genomic assembly metrics of strain BL-9^T^.

Scaffold Length (bp)	Scaffold Number	Scaffold N50 (bp)	Scaffold N90 (bp)	Contig Length (bp)	Contig Number	Contig N50 (bp)	Contig N90 (bp)	GC Content (%)	Gaps Number
5,516,319	17	807,666	213,912	5,515,736	18	807,666	213,912	49.78	1

**Table 2:** General genomic information of strain BL-9 and its closely related reference strains.

Strains	Accession No.	Genome Size (Mb)	DNA GC (%)	Gene Number	RNA		
					tRNA	Non-Coding rRNA	16S rRNAs
Test strain BL-9^T^	GCA_046559655.1	5.5	49.7	5081	91	6	1
Reference strain *Fontibacillus phaseoli* using the acetylene reduction assay	GCA_003337355.1	5.4	49.5	5071	74	4	9
Reference strain *Fontibacillus solani* A4STR04	GCA_014138385.1	5.5	50.5	5421	72	4	2
Reference strain *Paenibacillus segetis* DB13260	GCA_014639155.1	5.5	53.7	5126	72	4	5

**Table 3:** ANI and dDDH values between strain BL-9^T^ and its closely related species of *Fontibacillus* and *Paenibacillus* genera.

Species	GenBank Accession Number	ANI Value with Strain BL-9^T^	dDDH Value with Strain BL-9^T^
*Fontibacillus phaseoli* CECT 8333^T^ (=BAPVE7B)	GCA_003337355.1	90.94%	42.5%
*Fontibacillus solani* CECT 8693^T^	GCA_014138385.1	73.57%	19.2%
*Fontibacillus panacisegetis* DSM 28129^T^	GCA_900102215.1	73.46%	19.10%
*Fontibacillus* *aquaticus*	Data unavailable		
*Fontibacillus pullulanilyticus*	Data unavailable		
*Paenibacillus timonensis* DSM 16943^T^	GCA_022427145.1	74.25%	19.8%
*Paenibacillus rubinfantis* MT18^T^	GCA_001486505.1	74.10%	19.6%
*Paenibacillus macerans* NCTC6355^T^	GCA_900454495.1	74.81%	20.5%
*Paenibacillus vini* J42TS3	GCA_018403325.1	82.32%	25.7%
*Paenibacillus antibioticophila* DG11^T^	GCA_000455265.1	74.45%	20.2%
*Paenibacillus segetis* CGMCC1.12769^T^	GCA_014639155.1	72.40%	18.7%
*Paenibacillus anaericanus* DSM 15890^T^	GCA_003994475.1	72.30%	19.0%
*Paenibacillus solani* FJAT-22460^T^	GCa_001277345.1	70.31%	20.1%
*Paenibacillus zeisoli* 3-5-3^T^	GCA_003994465.1	71.16%	18.5%
*Paenibacillus shirakamiensis* DSM 26806^T^	GCA_017874255.1	69.72%	18.0%

**Table 4:** Differential characteristic features of strain BL-9^T^ from the reference strains.

Characteristic	Test Strain	Reference Strain	Reference Strain	Reference Strain
	BL-9^T^	*F. phaseoli* BAPVE7B^T^	*F. solani* A4STR04^T^	*P. segetis* DB13260^T^
Optimal pH range	7	7	7	8.5–9.0
Optimal NaCl	1.0%	0.5–1.0%	0.5–1.0%	0%
optimal Growth temperature (°C)	26	30	30	30–37
Gram reaction	−	+	+	+
Nitrate reduction	+	+	+	−
Starch hydrolysis	+	+	+	−
Mobility	+++	+	+	+
Flagellum	+	+	+	+
Production of acid from following substrates:				
D-fructose	+	−	+	+
D-glucose	+	+	−	+
D-galactose	+	+	w	+
Maltose	+	+	+	+
D-mannitol	+	−	−	+
D-sorbitol	+	−	−	+
Inositol	+	−	−	−
Sucrose	+	+	w	+
Lactose	+	+	+	+

+: positive; −: negative. w: weak reaction.

**Table 5:** The fatty acid contents of strain BL-9^T^ and its closely related reference strains.

Fatty Acid	Strain BL-9^T^	*F. phaseoli* BAPVE7B^T^	*F. solani* A4STR04^T^	*P. segetis* DB13260^T^
Saturated				
C_12:0_	0.29	1.6	0.5	1.8
C_14:0_	0.97	3.8	2.0	3.0
C_16:0_	13.83	18.3	12.3	13.5
C_17:0_	1.13	-	-	-
Unsaturated				
C_14:1_ ω5c	0.02	-	-	-
C_18:1_ ω9c	0.33	-	-	-
Branched saturated				
iso-C_14:0_	0.49	1.1	1.3	7.1
iso-C_15:0_	5.28	3.2	3.6	5.9
iso-C_16:0_	3.65	5.1	5.1	19.6
iso-C_17:0_	3.72	1.7	1.2	1.6
anteiso-C_15:0_	55.14	53.1	61.5	43.9
anteiso-C_17:0_	11.88	9.3	6.8	2.3

-: not detected.

## Data Availability

The original contributions presented in this study are included in the article/Supplementary Materials. Further inquiries can be directed to the corresponding author.

## Supplementary Materials

The following supporting information can be downloaded at: https://www.mdpi.com/article/10.3390/microorganisms14010049/s1, Figure S1: The maximum-likelihood phylogenetic tree based on *nifH* gene sequences (823 base pairs) of strain BL-9^T^ together with its closely related taxonomic groups. Bar, 0.05 nucleotide substitutions per site. Bootstrap values > 70% (based on 500 replications) are shown at branch points; Figure S2: Two-dimensional TLC plate of polar lipids extracted from strain BL-9^T^. The plate was sprayed with 10% (*v*/*v*) molybdophosphoric aicd to show all polar lipids present. DPG, diphosphatidylglycerol; PG, phosphatidylglycerol; APL, aminophospholipids; PL, unidentified phosphoglycolipids; L, unknown polar lipids; APGL, unidentified aminophosphoglycolipid; Figure S3: HPLC analysis shows MK-7 as the major respiratory quinone component for strain BL-9^T^. Mobile phase, methanol:isopropanol = 65:35; Chromatographic column: Zorbax Eclipse XDB-C18 (4.6 * 250 mm, 5 μm; Agilent); Column temperature: 40 °C; Flow rate: 1.0 mL/min; Injection volume: 10 μL; Detection wavelength: 270 nm; Table S1: Comparison of *nif* (*ni*trogen *f*ixation) genes from the novel species *Fontibacillus forbon *BL-9^T^ and *Fontibacillus phaseoli* BAPVE7B.
