# Supplementary material for: N2-Fixing Fontibacillus forbon sp. nov., a Novel Species from the Plant Rhizosphere
Source: Microorganisms. 2025 Dec 25;14(1):49. doi: 10.3390/microorganisms14010049 (PMC12843690; doi:10.3390/microorganisms14010049)
Supplement: Supplementary file 1 [file microorganisms-14-00049-s001.zip › microorganisms-4029366-supplementary.pdf]

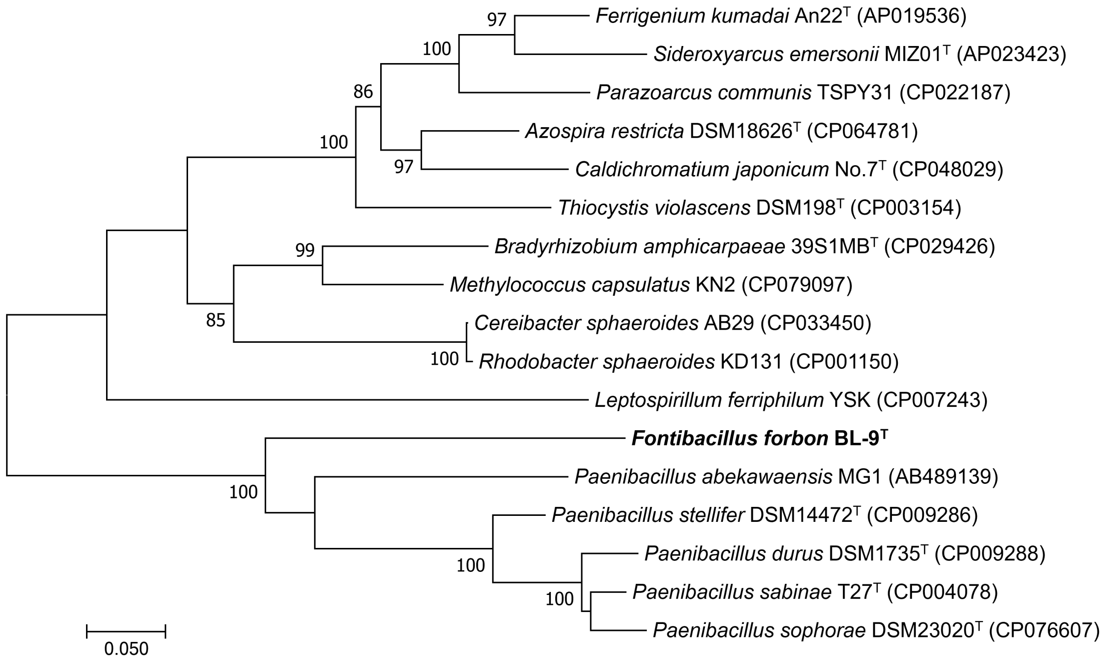

Fig. S1. The maximum-likelihood phylogenetic tree based on *nifH* gene sequences (823 base pairs) of strain BL-9^T together with its closely related taxonomic groups. Bar, 0.05 nucleotide substitutions per site. Bootstrap values>70% (based on 500 replications) are shown at branch points

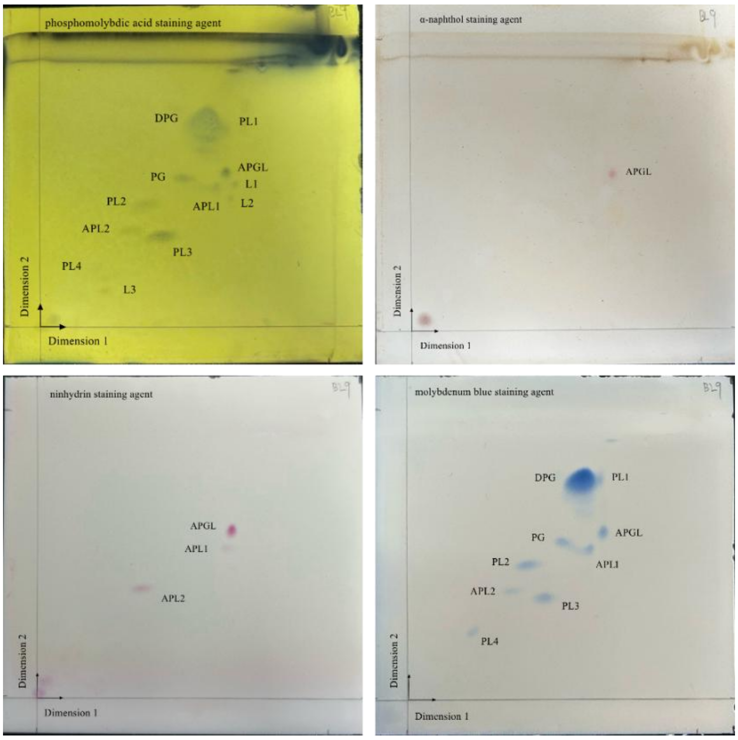

Fig. S2. Two-dimensional TLC plate of polar lipids extracted from strain BL-9^T. The plate was sprayed with 10% (v/v) molybdophosphoric aicd to show all polar lipids present. DPG, diphosphatidylglycerol; PG, phosphatidylglycerol; APL, aminophospholipids; PL, unidentified phosphoglycolipids; L, unknown polar lipids; APGL, unidentified aminophosphoglycolipid

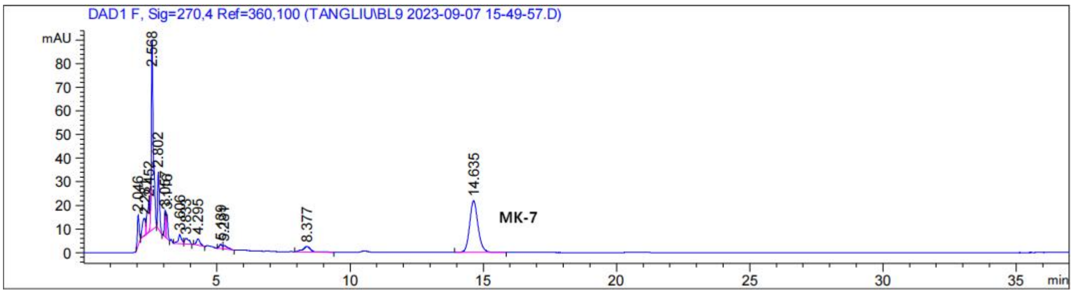

Fig. S3. HPLC analysis shows MK-7 as the major respiratory quinone component for strain BL-9^T. Mobile phase, methanol: isopropanol=65:35; Chromatographic column:
Zorbax Eclipse XDB-C18 (4.6 * 250 mm, 5 μ m; Agilent); Column temperature: 40 ℃; Flow rate:1.0 mL/min; Injection volume: 10 μ L; Detection wavelength: 270 nm

Table S1. Comparison of *nif* (*ni*trogen *fi*xation) genes from the novel species *Fontibacillus forbon* BL-9^T and *Fontibacillus phaseoli* BAPVE7B

Gene name	*Fontibacillus forbon* BL-9^T	*Fontibacillus phaseoli*	Identity
nifV	1146	1146	91.23%
hesA	756	756	92.99%
orf1	543	540	92.22%
nifX	414	414	92.27%
nifN	1326	1326	90.12%
nifE	1368	1368	92.91%
nifK	1536	1536	91.99%
nifD	1449	1449	95.93%
nifH	873	873	92.31%
nifB	1401	1392	93.25%
Total	10925	10928	91.52%
